# Supplementary material for: PolypNextLSTM: a lightweight and fast polyp video segmentation network using ConvNext and ConvLSTM
Source: Int J Comput Assist Radiol Surg. 2024 Aug 8;19(10):2111–9. doi: 10.1007/s11548-024-03244-6 (PMC11442634; doi:10.1007/s11548-024-03244-6)
Supplement: Supplementary file 1 — (pdf 2042 KB) [file 11548_2024_3244_MOESM1_ESM.pdf]

Supplementary material for PolypNextLSTM: A lightweight and fast polyp video segmentation network using ConvNext and ConvLSTM

Debayan Bhattacharya[1†], Konrad Reuter[1*†], Finn Behrendt[1], Lennart Maack[1], Sarah Grube[1], Alexander Schlaefer[1]

[1]Institute of Medical Technology and Intelligent Systems, Technische Universitaet Hamburg, Hamburg, Germany.

*Corresponding author(s). E-mail(s): konrad.reuter@tuhh.de;
†These authors contributed equally to this work.

Abstract

This document serves as the supplementary material to the main paper titled "PolypNextLSTM: A lightweight and fast polyp video segmentation network using ConvNext and ConvLSTM"

Keywords: video, polyp, segmentation, CNN

1 Experiments with 'Seen' test set configuration

Tables 1 display the comprehensive performance evaluations of various methods on SUN-SEG-Easy test sets, categorized as 'Easy Seen' and 'Hard Seen'. Our model consistently surpasses all comparative models across all metrics, including 'seen' and 'unseen' scenarios. As expected, 'seen' cases generally yield higher overall metrics in both 'Easy' and 'Hard' scenarios.

In Table 1, our *PolypNextLSTM* demonstrates superior performance, with a Dice increase of +0.0219 (+2.53%) compared to the second-best model, SANet—an image-based approach.

		Easy Seen				Hard Seen					
		Dice	IOU	HD95	Recall	Dice	IOU	HD95	Recall	Params	FPS
**Image**	DeepLab [1]	0.8538	0.7821	7.497	0.8270	0.8298	0.7472	11.75	0.7925	39.63M	54
	PraNet [2]	0.8632	0.8009	7.300	0.8552	0.8432	0.7713	9.590	0.8236	32.55M	45
	SANet [3]	0.8643	0.7967	7.810	0.8492	0.8444	0.7695	10.90	0.8185	23.90M	71
	TransFuse [4]	0.8370	0.7675	9.696	0.8234	0.8060	0.7285	13.35	0.7911	26.27M	63
	CASCADE [5]	0.8618	0.7981	7.574	0.8554	0.8360	0.7629	10.90	0.8203	35.27M	54
**Video**	COSNet [6]	0.8212	0.7492	10.30	0.8158	0.8016	0.7229	12.87	0.7742	81.23M	16
	HybridNet [7]	0.8462	0.7668	7.337	0.8559	0.8140	0.7288	9.735	0.8126	101.5M	67
	PNSNet [8]	0.8513	0.7864	7.527	0.8332	0.8295	0.7564	9.755	0.7989	26.87M	61
	PNS+ [9]	0.8590	0.7980	7.212	0.8545	0.8336	0.7650	10.02	0.8136	26.87M	57
	SSTAN [10]	0.8517	0.7856	8.655	0.8436	0.8328	0.7588	12.36	0.8215	30.15M	101
	**Ours**	**0.8862**	**0.8272**	**5.808**	**0.8843**	**0.8643**	**0.7948**	**8.802**	**0.8532**	**21.95M**	**108**

Table 1 Comparison to various state-of-the-art models on the seen cases. The top five models are image models, while the bottom five are video-based models.

We also present the Dice score results categorized by visual attributes in Table 2 for the 'Easy Seen' test set and in Table 3 for the 'Hard Seen' test set. In the 'Easy Seen' set, our model excels in multiple attributes excluding FM, OV and SV. While our model performs competitively in other categories, the largest margin appears in IB, where PNS+ outperforms by +0.0283 (+3.77%). In the 'Hard Unseen' test set, our model emerges as the top performer across most categories. Particularly noteworthy is the substantial improvement in IB again, showcasing +0.0302 (+3.47%) compared to the second-best model (CASCADE). Given the generally lower scores, IB stands out as the most challenging category in 'Seen' test set as well.

		SI	IB	HO	GH	FM	SO	LO	OCC	OV	SV
**Image**	DeepLab[1]	0.7534	0.6033	0.9359	0.8260	0.7779	0.8298	0.9005	0.8497	0.7907	0.7728
	PraNet[2]	0.8115	0.7003	0.9508	0.8120	0.7568	0.8275	0.9176	0.8808	0.7958	0.7659
	SANet[3]	0.8001	0.7294	0.9295	0.8272	0.7420	0.8516	0.8897	0.8759	0.7882	0.7733
	TransFuse[4]	0.6923	0.6151	0.8901	0.8036	**0.7874**	0.8178	0.8609	0.8157	0.7829	0.7383
	CASCADE[5]	0.7972	0.7231	0.8787	0.8336	0.7858	0.8607	0.8417	0.8743	0.7548	**0.7956**
**Video**	COSNet[6]	0.7139	0.6080	0.8842	0.7798	0.7541	0.7844	0.8542	0.8266	0.7518	0.7504
	HybridNet[7]	0.7962	0.7169	0.9226	0.8111	0.7592	0.8193	0.8755	0.8745	0.7638	0.7757
	PNSNet[8]	0.8368	0.7420	0.9361	0.7784	0.7388	0.8157	0.8981	0.8937	0.7704	0.7523
	PNS+[9]	0.8501	0.7505	0.9341	0.7994	0.7722	0.8218	0.8929	0.9015	**0.8005**	0.7862
	SSTAN[10]	0.7814	0.6928	0.9479	0.8032	0.7513	0.8093	0.9110	0.8609	0.7873	0.7746
	**Ours**	**0.8574**	**0.7788**	**0.9643**	**0.8379**	0.7616	**0.8691**	**0.9274**	**0.9040**	0.7992	0.7872

Table 2 Comparison of the dice score divided by the visual attributes occurring in the clips of the "Easy Seen" test set. The best score for each category is marked in bold.

		SI	IB	HO	GH	FM	SO	LO	OCC	OV	SV
**Image**	DeepLab[1]	0.8034	0.8171	0.7703	0.8313	N/A	0.8116	0.7703	0.8565	0.7741	0.7985
	PraNet[2]	**0.8308**	0.8652	**0.8046**	0.8296	N/A	0.8123	**0.8046**	0.8703	**0.8093**	0.8133
	SANet[3]	0.8108	0.8703	0.7900	0.8402	N/A	0.8261	0.7900	0.8500	0.7872	0.7854
	TransFuse[4]	0.7543	0.7711	0.7142	0.8169	N/A	0.8127	0.7142	0.8599	0.7346	0.8105
	CASCADE[5]	0.7973	0.8707	0.7178	0.8383	N/A	0.8411	0.7178	0.8614	0.7419	**0.8192**
**Video**	COSNet[6]	0.7709	0.8269	0.7086	0.7907	N/A	0.7922	0.7086	0.8378	0.7206	0.7790
	HybridNet[7]	0.7806	0.8648	0.7819	0.8028	N/A	0.7789	0.7819	0.8354	0.7630	0.7435
	PNSNet[8]	0.8120	0.8559	0.7748	0.8133	N/A	0.8058	0.7748	0.8670	0.7882	0.8144
	PNS+[9]	0.8218	0.8568	0.7748	0.8213	N/A	0.8003	0.7748	0.8683	0.7898	0.8140
	SSTAN[10]	0.8035	0.8471	0.7872	0.8290	N/A	0.8100	0.7872	0.8677	0.7919	0.7996
	**Ours**	0.8301	**0.9005**	0.7903	**0.8606**	N/A	**0.8535**	0.7903	**0.8736**	0.7883	0.8174

Table 3 Comparison of the dice score divided by the visual attributes occurring in the clips of the "Hard Seen" test set. The best score for each category is marked in bold. The "Hard Seen" test set does not contain samples for the fast motion category, therefore it is empty.

2 Ablation Study

2.1 Backbone comparison without temporal fusion module

To show how much value the temporal fusion module adds to the model, a version without the temporal fusion modules is trained. Furthermore, different backbones for the base model are compared. To be more specific, the base model is trained with four different backbones:

- Reduced ConvNext-tiny (our model of choice)
- Full ConvNext-tiny
- Reduced SwinV2-tiny
- Full SwinV2-tiny

We trained a base model using the full ConvNext-tiny as its backbone to measure the accuracy impact upon removing the last processing stage. Additionally, we trained models with both full and reduced SwinV2-tiny [11] backbones. Here, 'reduced' signifies the removal of not only the classification layers but also the last processing stage, known for its higher parameter count. The selection of SwinV2-tiny for comparison stemmed from its performance proximity to ConvNext-tiny on the ImageNet1k dataset [12], as observed in the performance metrics provided by PyTorch/Torchvision [13]. Table 4 presents a snapshot from the overall comparison, highlighting the higher computational cost associated with the Swin model compared to the ConvNext model.

Model	Acc@1	Acc@5	Params	GFLOPS
ConvNext-tiny	82.520	96.146	28.6M	4.46
SwinV2-tiny	82.072	96.132	28.4M	5.96

Table 4 Comparison of the two considered backbones from [13]. Acc@1 and Acc@5 stands for top 1 and top 5 accuracy on the ImageNet1k dataset.

In Table 5, a comparison is presented among base models employing four different backbones. Here, 'base model' refers to an encoder-decoder structure without temporal fusion modules, with our proposed model utilizing the reduced ConvNext-tiny backbone. Our model's results are appended as the final row for comparison. Notably, the ConvNext backbone consistently outperforms its SwinV2 counterpart. Specifically, the full ConvNext-tiny backbone surpasses the full SwinV2-tiny, and similarly, the reduced ConvNext-tiny outperforms the reduced SwinV2-tiny. Intriguingly, the results showcase that the reduced ConvNext-tiny backbone achieves performance levels comparable to those of the full SwinV2-tiny backbone.

Moreover, comparing these results to our proposed model utilizing the bidirectional ConvLSTM reveals significant overall improvement, emphasizing the substantial advantage conferred by the temporal fusion module over base models.

	Easy Unseen				Hard Unseen					
	Dice	IOU	HD95	Recall	Dice	IOU	HD95	Recall	Params	FPS
SwinV2-tiny	0.7304	0.6493	21.99	0.6822	0.7257	0.6422	21.42	0.6919	34.38M	71
reduced SwinV2-tiny	0.7052	0.6260	21.88	0.6573	0.6921	0.6095	21.19	0.6564	13.83M	85
ConvNext-tiny	0.7360	0.6615	20.03	0.6952	0.7508	0.6717	17.89	0.7238	34.62M	112
reduced ConvNext-tiny	0.7264	0.6514	18.32	0.6796	0.7340	0.6552	16.77	0.6975	13.99M	135
**Ours**	**0.7686**	**0.6958**	**15.91**	**0.7350**	**0.7838**	**0.7067**	**14.07**	**0.7641**	21.95M	108

Table 5 Results of the base model using different backbone networks for the unseen cases.

2.2 Bidirectional vs Unidirectional ConvLSTM

To validate the efficacy of the bidirectional ConvLSTM model, we conducted a comparison with a model utilizing only a unidirectional ConvLSTM. Table 6 presents the results of this comparison. While the unidirectional ConvLSTM demonstrates some improvement over the base model, the magnitude of enhancement is relatively modest. In contrast, the bidirectional approach used in *PolypNextLSTM* approach yields a substantial performance boost, underscoring the advantage of bidirectional temporal feature flow for optimal performance.

	Easy Unseen				Hard Unseen					
	Dice	IOU	HD95	Recall	Dice	IOU	HD95	Recall	Params	FPS
Base Model	0.7264	0.6514	18.32	0.6796	0.7340	0.6552	16.77	0.6975	13.99M	135
Unidirectional ConvLSTM	0.7318	0.6591	18.00	0.6889	0.7451	0.6660	16.22	0.7124	24.60M	108
**Ours**	**0.7686**	**0.6958**	**15.91**	**0.7350**	**0.7838**	**0.7067**	**14.07**	**0.7641**	21.95M	108

Table 6 Comparison of the results using a single direction ConvLSTM vs. using a bidirectional ConvLSTM for the unseen cases.

2.3 Choice of Temporal Fusion Module

To prove the effectiveness of the bidirectional ConvLSTM as the module for temporal fusion, four popular temporal fusion approaches are investigated. The modules are designed in a way, that they can directly replace the bidirectional ConvLSTM in our proposed model.

2.3.1 Channel Stacking

A straightforward approach involves stacking encoded images along the channel dimension and subsequently passing them through 2D convolutions, as illustrated in Figure 1. This method employs two 2D convolutions, each followed by batch normalization and ReLU activation. Both convolutional layers use a 3×3 kernel size and 1×1 padding to maintain the input dimension. For an input shape of $F \times C \times H \times W$ (where F denotes the number of frames, C the channels, H the image height, and W the width), stacking the images along the channel dimension generates a tensor of $F \cdot C \times H \times W$. The first convolution compresses the channel dimension by a factor of F, resulting in a shape of $C \times H \times W$. Subsequently, the second convolution expands the channel dimension back to $F \cdot C \times H \times W$. This reduction is crucial for limiting parameters, yet it may lead to information loss. To mitigate this, a skip connection spanning the entire module is introduced. Reformatting the data restores the input dimension to $F \times C \times H \times W$.

2.3.2 3D convolutions

Utilizing 3D convolutions offers a means to integrate temporal information into convolutions without requiring a data reshaping process. This approach aligns with the methodology employed in the Hybrid2D/3D network presented in [7]. Similar to the channel stacking method, our approach incorporates two 3D convolutions, each followed by batch normalization and ReLU activation functions. A kernel size of 3 and padding of 1 are applied to maintain the input dimensions. Notably, this varies from the Hybrid2D/3D network approach, where the kernel size and padding were adjusted to reduce the frame dimension (F) to 1. A visual representation of this process is depicted in Figure 2. The module begins by swapping the frame dimension with the channel dimension to align with the input format expected by the 3D convolution layer. Eventually, the channels are swapped back to restore the initial input dimension.

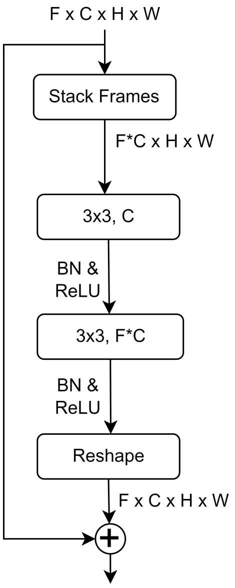

Fig. 1 Visualization of the channel stacking module. F is the number of frames, C the number of channels and H&W the height and width.

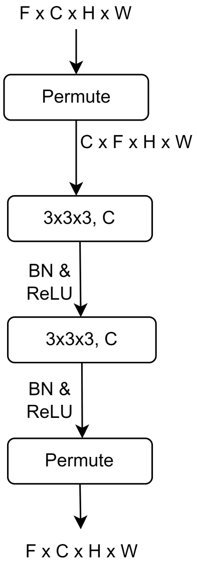

Fig. 2 Visualisation of the 3D convolution approach. Again, F is the frame dimension, C the number of channels and H&W are the height and width.

2.3.3 Multi-Headed Attention

In [10], the utilization of two Multi-Head Attention modules was proposed to incorporate both temporal and spatial attention into the model. We adopt a similar approach, implementing a module that uses Multi-Head Attention (MHA) specifically along the temporal dimension to interrelate frames. However, for spatial attention, we adopt the Shifted Window (Swin) Multi-Head Attention V2 module from the SwinV2 transformer [11]. This adaptation enhances spatial attention efficiency, particularly for

higher image resolutions. To facilitate a more localized operation, a secondary Swin attention module establishes connections between restricted regions, mirroring the strategy in SwinV2 transformers. Additionally, linear feed-forward layers are incorporated after the attention modules, maintaining skip connections between sub-modules to mimic transformer structures. The structure is illustrated in Figure 3. Both Swin attention blocks employ four heads and a window size of 8×8. The first block operates without shifts, while the second shifts windows by 4×4. Two linear layers form the feed-forward layers, expanding the channel dimension by 4 and then reducing it back to the initial input channels, following the transformer pattern. ReLU activation functions follow each linear layer, with layer normalization applied after each module (Attention or Linear Layers).

This module involves multiple reshapes and dimension swaps to align with the correct input dimensions for sub-modules, which are not shown in the figure for simplicity. Instead, the input dimensions for each sub-module are specified. The temporal multi-attention block requires data in the shape $H \cdot W \times F \times C$, considering each pixel in the input as an embedded patch with C as the patch's embedding dimension and F as the sequence length for attention calculation. For the linear layers, the data can revert to being four-dimensional, with the channel dimension as the last. The shifted window attention module requires data in the shape $F \times H \times W \times C$, enabling independent spatial attention computation for each frame.

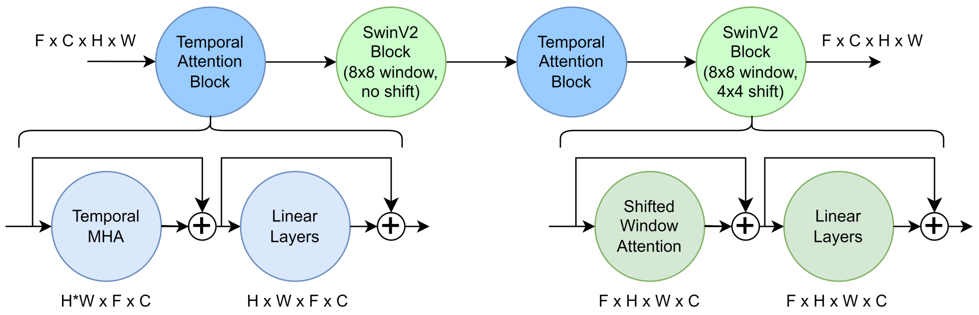

Fig. 3 Visualization of the Attention based module for including temporal information. The input shape for the different submodules is given. F is the number of frames, C the channels and H&W the height and width.

2.3.4 Normalized Self-Attention (NSA) Block

We employ the Normalized Self-Attention Block (NS-Block) from PNSNet [8] and PNS+ [9] for integrating temporal information. This block applies a locally constrained spatio-temporal multi-head attention mechanism. Preceding the NS-Blocks, the PNSNet/PNS+ integrates a receptive field block (RFB) [14] to enhance backbone-extracted features and diminish the channel dimension, thereby reducing the computational load for the NS-Block. Adapting this method, we insert an RFB ahead of the NS-Blocks. Configured to compress the channel dimension by a factor of 8, the

RFB (depicted in Figure 4) operates via a sequence: first passing through a squeeze-excitation block (visualized in Figure 5), then dividing into four branches utilizing distinct kernel sizes and dilation rates for varied scale information capture (except for the 1x1 convolutions, which employ depthwise convolutions).

The module overview (illustrated in Figure 6) deployed in this study starts with the RFB, followed by two consecutive NS-Blocks interconnected by skip connections, akin to PNSNet/PNS+. Employing the same configuration as in [9], the NS-Blocks use a kernel size of 3 and 4 groups (heads). Dilation rates for the first NS-Block are set to (3, 4, 3, 4), and for the second, they are (1, 2, 1, 2). Ultimately, a linear layer restores the input shape. Similarly, to counteract potential information loss due to channel dimension reduction from the RFB, we introduce a skip connection covering the entire module, akin to the channel stacking approach.

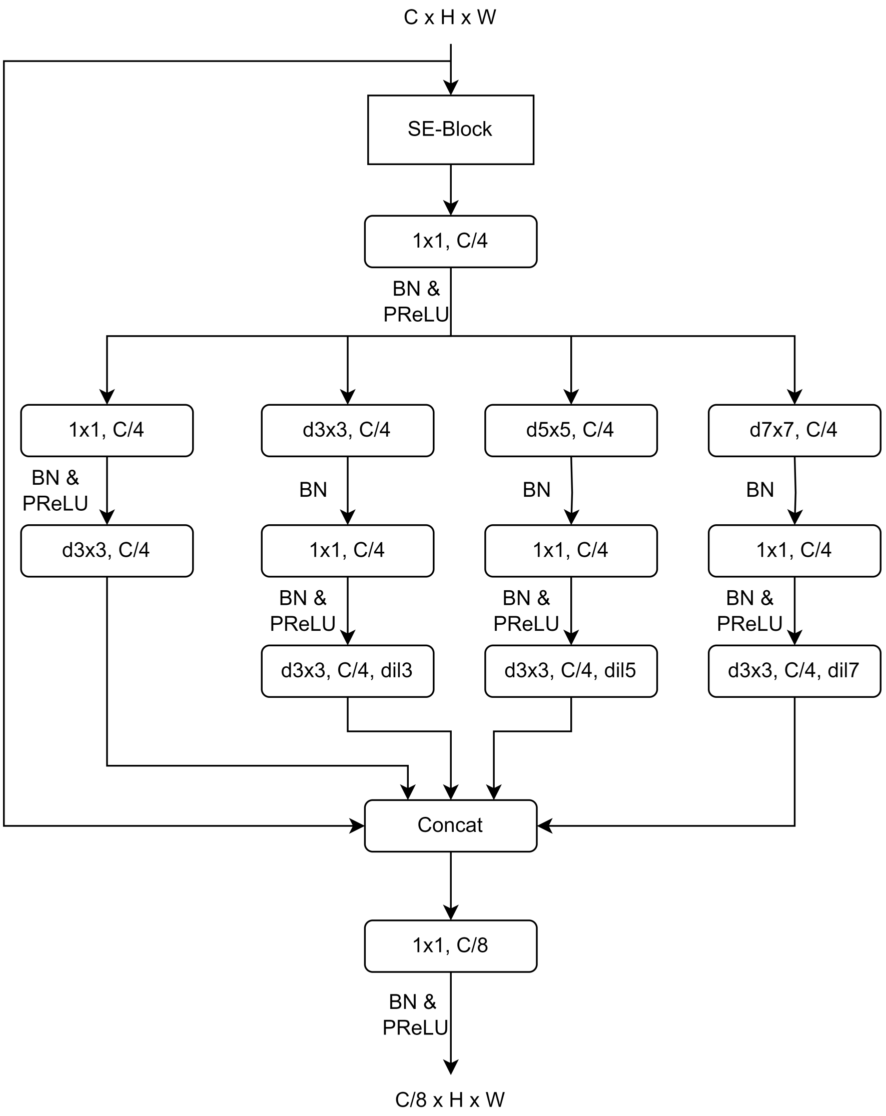

Fig. 4 The complete structure of the receptive field block utilized in the NSA module. The input is passed through a squeeze-excitation block (visualized in Figure 5 and afterwards passed through four different paths using depthwise convolutions to capture context with different receptive fields. The results are concatenated and passed through a final convolution.

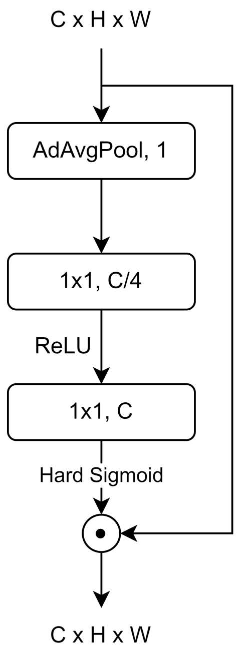

Fig. 5 Visualization of the SE-Block utilized in the receptive field block.

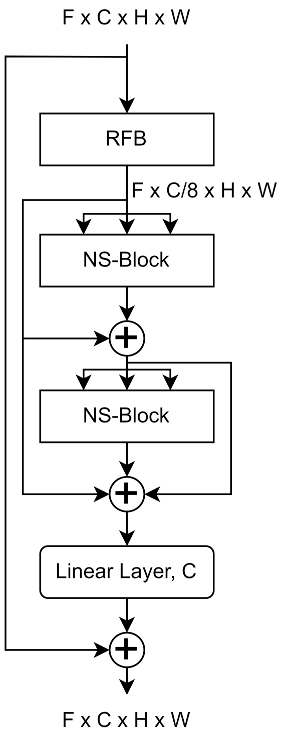

Fig. 6 Visualization of the NS-Block based module. The input is passed through a RFB and afterwards through two successive NS-Blocks. A linear layer is added to restore the number of input channels. Furthermore, various skip connections are added.

The results are depicted in Table 7. Across all temporal fusion methods, enhancements over the base model are evident. However, the 3D convolutional approach shows marginal improvement, with metrics closely aligning with the base model. Notably, our model outperforms all other methodologies across all evaluated metrics, except for the Hausdorff distance in the hard unseen dataset, where the channel stacking method displayed superior results. The channel stacking technique consistently performs well and stands out as the second-best temporal fusion module in this context.

The findings presented in Table 7 affirm the effectiveness of temporal fusion module. All temporal fusion techniques exhibit performance improvement with a single fusion module. However, none achieve a performance comparable to our proposed model. Surprisingly, the channel stacking method, despite its simplicity, attains commendable performance, outperforming most considered state-of-the-art models. Its challenge may arise with larger input frames, where the convolutional layers would demand more parameters, risking information loss due to compressed channel dimensions. With 27.26 million parameters, it's already the largest model among these methods.

The 3D convolutional approach marginally enhances metrics compared to the base model. However, its limitation lies in the two successive 3D convolutions with a kernel size of 3, capturing only a limited number of frames for each output frame. To address this, a potential solution is using a kernel size of $5 \times 3 \times 3$, ensuring consideration of all input frames for each output, albeit resulting in significantly more parameters in these layers. In contrast, the HybridNet [7] employs two 3D convolutions reducing the temporal dimension to one, a strategy that increases parameters less steeply for longer input sequences, albeit producing a single channel feature map per input sequence.

Approaches using temporal attention and NS-Blocks exhibit promising results, albeit falling short compared to the channel stacking method. Interestingly, our NS-Block-based model surpasses the PNSNet [8] and PNS+ [9], indicating the efficiency of ConvNext-tiny as a backbone.

The NSA approach offers competitive results, running at 74 frames per second (FPS) but with the least trainable parameters (14.22 million), marginal increase over the base model parameter count (13.99 million). Attention-based approaches and ConvLSTM-based models hold an advantage—they adapt effortlessly to varying input sequence lengths without altering the parameters, unlike convolution-based methods that increase parameters with input sequence length, hindering speed and training ease.

This approach proves effective for most temporal fusion modules, aligning with the concept of independently encoding images and forming connections on fully encoded states, a strategy common in four of the five compared video-based models [6–9].

	Easy Unseen				Hard Unseen					
	Dice	IOU	HD95	Recall	Dice	IOU	HD95	Recall	Params	FPS
Base Model	0.7264	0.6514	18.32	0.6796	0.7340	0.6552	16.77	0.6975	13.99M	135
Channel Stacking	0.7543	0.6824	16.86	0.7182	0.7608	0.6846	**14.06**	0.7391	27.26M	125
3D Convolutions	0.7292	0.6558	18.24	0.6850	0.7368	0.6577	16.38	0.7012	21.95M	115
MHA	0.7382	0.6666	17.82	0.7000	0.7482	0.6730	15.50	0.7219	21.09M	105
NSA	0.7429	0.6691	18.39	0.7024	0.7569	0.6793	16.03	0.7298	14.22M	74
**Ours**	**0.7686**	**0.6958**	**15.91**	**0.7350**	**0.7838**	**0.7067**	14.07	**0.7641**	21.95M	108

Table 7 Experiment 1, unseen cases: Average results of the cross validation from experiment 1 on the unseen polyp cases. The best performing model for each metric is marked in bold.

2.4 Placement of Temporal Fusion Module in the skip connections

Incorporating additional temporal fusion modules at skip connections, as illustrated in Figure 8, retains the original backbone network structure without modification. This strategy aims to obtain more detailed predictions by extending the temporal fusion to higher resolutions through multiple skip connections.

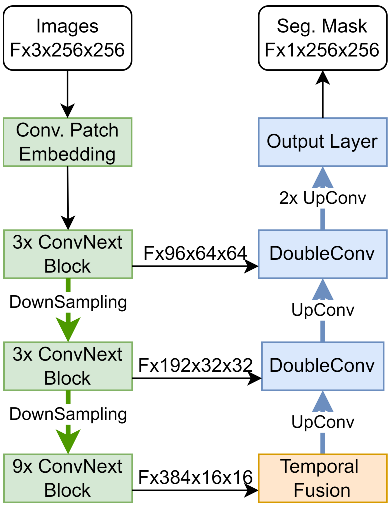

Fig. 7 Visualization of the approach to include temporal information into the base network. A temporal fusion module is placed only after the last encoder stage and before the decoding begins.

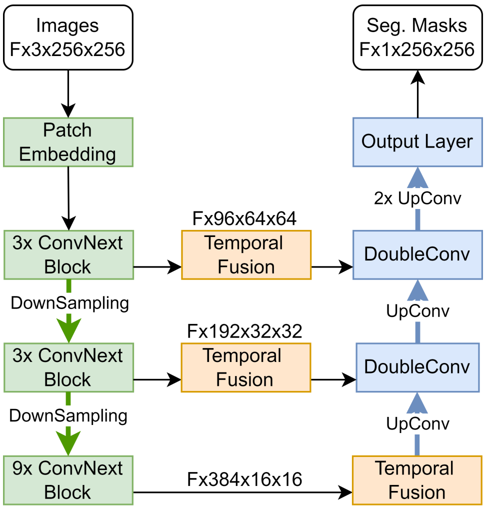

Fig. 8 Visualization of the approach for including temporal information. Additionally to the temporal fusion block in the first experiment there are also temporal fusion modules at the skip connections.

The results are depicted in Table 8. Once more, all temporal fusion modules exhibit improvements over the base model, particularly demonstrating enhanced performance on the 'hard' dataset. In comparison, our model excels in dice, IOU, and recall. Nevertheless, this time, the temporal attention approach yields the best results for HD95 across both test sets. Notably, the MHA approach and the 3D convolutions exhibit more pronounced improvements in this model configuration than in the approach of placing the temporal fusion module only in the bottleneck, showcasing enhanced performance. The improved HD95 metric may be attributed to the fact that edge alignment of predicted and ground truth mask is better due to the temporal processing at high resolutions. Surprisingly, the ConvLSTM model, which integrates additional bidirectional ConvLSTMs at skip connections, results in a decrement in performance. Due to the introduction of additional temporal fusion modules, the models generally exhibit slower processing speeds compared to the first approach. Despite this, all models outperform the base model, indicating the viability of this general approach. Notably, for the channel stacking approach, the incorporation of extra temporal fusion modules at skip connections leads to slightly inferior results (-0.0172 (2.28%) / -0.0124 (1.63%) dice for the easy/hard test set). A similar impact is observed for the ConvLSTM approach, essentially our proposed model with added bidirectional ConvLSTMs at skip connections (-0.0273 (3.55%) / -0.0348 (4.44%) dice on the easy/hard test set). The utilization of skip connections typically offers enhanced gradient flow through network layers, yet the addition of modules might disrupt this gradient flow, affecting performance. Surprisingly, the 3D convolution approach showcases substantial improvement

with added modules at skip connections, achieving state-of-the-art performance. Both attention-based approaches also display enhanced performance. Particularly, the MHA method demonstrates impressive results, outperforming other state-of-the-art methods. However, the increased computational burden and slower processing speeds limit its immediate application. This approach underscores the choice of shifted window attention over regular multi-head attention for the spatial dimension, as it avoids excessive computational demands.

	Easy Unseen				Hard Unseen					
	Dice	IOU	HD95	Recall	Dice	IOU	HD95	Recall	Params	FPS
Base Model	0.7264	0.6514	18.32	0.6796	0.7340	0.6552	16.77	0.6975	13.99M	135
Bi. ConvLSTM	0.7413	0.6688	18.42	0.7029	0.7490	0.6690	16.92	0.7177	24.44M	81
Channel Stacking	0.7371	0.6655	17.51	0.6958	0.7484	0.6724	14.75	0.7229	31.41M	114
3D Convolutions	0.7558	0.6825	17.44	0.7140	0.7598	0.6823	15.02	0.7318	24.44M	96
MHA	0.7635	0.6920	**15.45**	0.7287	0.7754	0.7000	**13.40**	0.7547	23.33M	55
NSA	0.7503	0.6774	17.83	0.7104	0.7675	0.6898	15.21	0.7389	14.47M	30
**Ours**	**0.7686**	**0.6958**	15.91	**0.7350**	**0.7838**	**0.7067**	14.07	**0.7641**	21.95M	108

Table 8 Experiment 2, unseen cases: Average results of the cross validation from experiment 2 on the unseen polyp cases. The best performing model for each metric is marked in bold.

2.5 Placement of Temporal Fusion Module after every encoder block

In this strategy, we integrate temporal fusion directly into the encoder structure. Post each encoder block, a dedicated temporal fusion module is inserted, leveraging temporal information before downsampling the data. The objective behind integrating this fusion mechanism directly within the encoder is to amplify the quality of extracted features at each encoding stage, ultimately aiming for enhanced overall performance. The schematic representation of this approach is depicted in Figure 9.

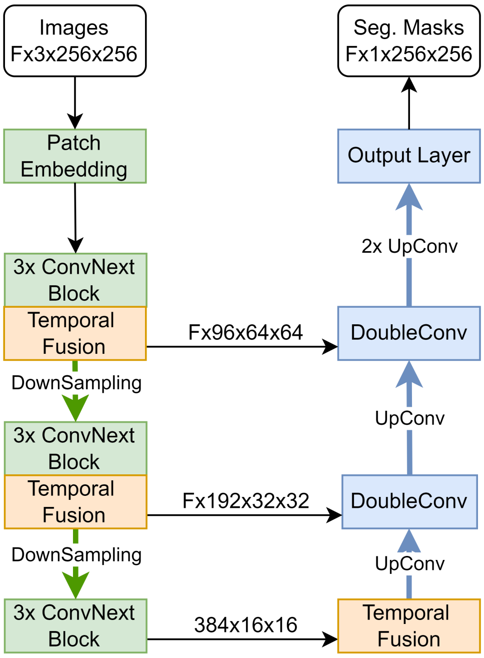

Fig. 9 The third approach for including temporal information. Additionally to the temporal fusion modules from experiment 1 there are temporal fusion modules after the encoding blocks to include temporal information already during encoding.

The results for this model configuration, where temporal fusion modules are directly embedded within the encoder, are presented in Table 9. Surprisingly, apart from the channel stacking approach that displays slight improvements, the other models demonstrate a notable decline in performance across most metrics. However, while the channel stacking approach exhibits promising results on the 'Easy' dataset, its performance on the 'Hard' dataset significantly lags behind our proposed model. Typically, the models from the third approach are performing suboptimally on the 'hard' test set.

	Easy Unseen				Hard Unseen					
	Dice	IOU	HD95	Recall	Dice	IOU	HD95	Recall	Params	FPS
Base Model	0.7264	0.6514	18.32	0.6796	0.7340	0.6552	16.77	0.6975	13.99M	135
Bi. ConvLSTM	0.7245	0.6496	20.65	0.6900	0.7040	0.6262	20.86	0.6865	24.44M	81
Channel Stacking	0.7544	0.6820	16.02	0.7189	0.7413	0.6671	15.52	0.7151	31.41M	114
3D Convolutions	0.6896	0.6179	22.55	0.6491	0.6582	0.5846	23.50	0.6329	24.44M	96
MHA	0.7044	0.6255	22.95	0.6630	0.6693	0.5923	23.24	0.6446	23.33M	55
NSA	0.6792	0.6026	24.86	0.6412	0.6532	0.5762	24.38	0.6290	14.47M	30
**Ours**	**0.7686**	**0.6958**	**15.91**	**0.7350**	**0.7838**	**0.7067**	**14.07**	**0.7641**	21.95M	108

Table 9 Experiment 3, unseen cases: Average results of the cross validation from experiment on the seen polyp cases. The best performing model for each metric is marked in bold.

This approach demonstrates limited success, where only the channel stacking module exhibits enhanced performance compared to the base model. Both this approach and the temporal fusion at skip connection share identical components arranged differently, resulting in matching speeds and parameter counts. We conjecture the core

issue seems to arise from introducing new, untrained layers into a pre-trained backbone, causing weight interference and resulting in suboptimal outcomes. However, the channel stacking approach might still fare better due to its skip connection allowing an alternative gradient path. It contrasts with the NSA module, which also utilizes a skip connection but relies on more complex architecture.

Generally, amending the carefully crafted backbone architecture by adding intermediate layers might not be a favourable strategy. However, employing a different training strategy could potentially yield better results. For instance, freezing the ConvNext layers and initially training the remaining network for a few epochs could allow the temporal modules to adapt to the ConvNext model's pre-trained weights before fine-tuning the entire model.

In summary, both the approaches described in Section 2.8 and Section 2.9 showcase performance enhancements across various temporal fusion modules. None of these models outperform our proposed model. While the MHA method described in Section 2.8 comes close, it is comparatively less computationally efficient and has a low FPS.

2.6 Backbone comparison with temporal fusion module

For further insights into how the removal of the last processing stage from the ConvNext-tiny backbone impacts the final model, we compare *PolypNextLSTM* to a model with similar configuration but using a full ConvNext-tiny backbone. The results are presented in Table 10. Interestingly, our proposed model performs slightly better than the model using the full ConvNext backbone. A reason for this could be that using the full backbone leads to a different input dimension for the bidirectional ConvLSTM module (8×8 instead of 16×16). The reduction in spatial dimension might also lead to a loss of spatial information and therefore result in slightly worse performance. Considering that pruning the backbone does not only makes the model faster but also increases the accuracy validates our decision to reduce the backbone model.

	Easy Unseen				Hard Unseen					
	Dice	IOU	HD95	Recall	Dice	IOU	HD95	Recall	Params	FPS
PolypNextLSTM unpruned	0.7599	0.6849	19.15	0.7203	0.7779	0.6992	16.74	0.7531	66.47M	72
**PolypNextLSTM (Ours)**	**0.7686**	**0.6958**	**15.91**	**0.7350**	**0.7838**	**0.7067**	**14.07**	**0.7641**	**21.95M**	**108**

Table 10 Comparison of our proposed *PolypNextLSTM* to a model with similar configuration but using the full (unpruned) ConvNext-tiny backbone.

2.7 Number of Frames

We investigate the impact of varying input frames on our proposed *PolypNextLSTM*. Figure 10 and Table 11 showcase the results across different metrics concerning the number of frames for the unseen test set configurations. *PolypNextLSTM* exhibit their poorest performance with one or two input frames, gradually improving up to five frames where a distinct performance peak emerges across all metrics. Beyond five frames, there is a noticeable decline in results. Hence, empirically, processing five frames emerges as the optimal configuration where *PolypNextLSTM* delivers its peak performance.

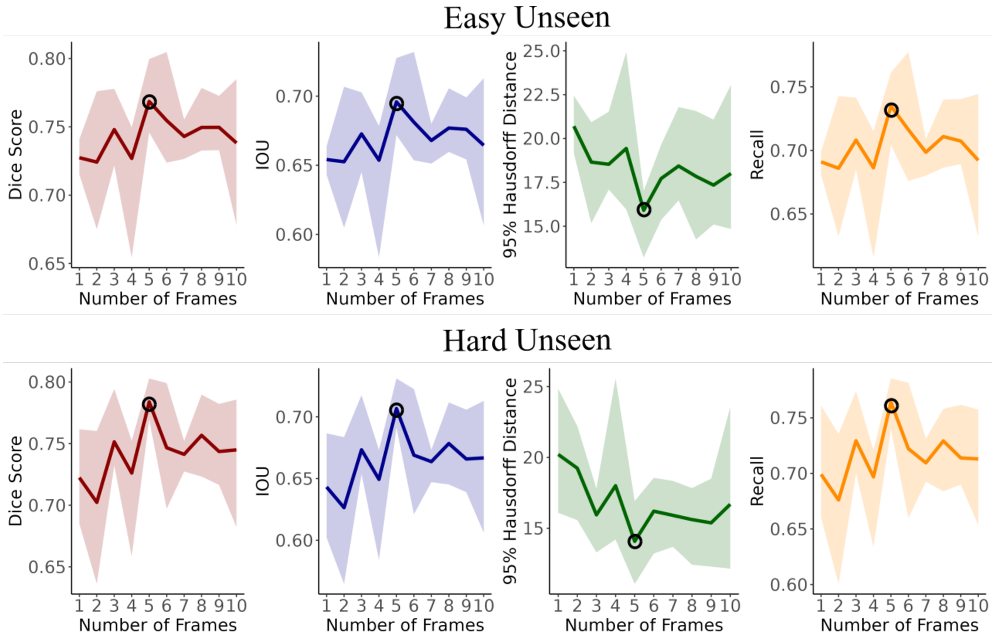

Fig. 10 Variation of the number of frames for the different test set configurations for four different metrics. 'Number of Frames' refers to how many images are processed simultaneously by the network. For dice score, IOU and recall, higher values indicate better performance. For the Hausdorff distance lower values refer to a better performance. The coloured interval refers to the minimum and maximum of the cross-validation. Black circle shows the highest metric.

	Easy Unseen				Hard Unseen			
Frames	Dice	IOU	HD95	Recall	Dice	IOU	HD95	Recall
1	0.7274	0.6541	20.70	0.6910	0.7224	0.6432	20.21	0.6992
2	0.7242	0.6526	18.65	0.6859	0.7023	0.6264	19.24	0.6761
3	0.7480	0.6727	18.53	0.7081	0.7514	0.6733	15.95	0.7293
4	0.7269	0.6537	19.43	0.6864	0.7261	0.6494	18.00	0.6969
5	**0.7686**	**0.6958**	**15.91**	**0.7350**	**0.7838**	**0.7067**	**14.07**	**0.7641**
6	0.7547	0.6812	17.72	0.7163	0.7467	0.6690	16.20	0.7222
7	0.7430	0.6679	18.43	0.6985	0.7413	0.6637	15.91	0.7095
8	0.7496	0.6769	17.84	0.7109	0.7567	0.6784	15.61	0.7292
9	0.7497	0.6760	17.35	0.7074	0.7436	0.6659	15.38	0.7139
10	0.7382	0.6644	18.00	0.6922	0.7449	0.6668	16.69	0.7130

Table 11 Results for the frame variation experiment on the unseen test sets. The best result for each metric is marked in bold.

Varying the number of frames indicates suboptimal results for sequences of one or two frames. This implies crucial information spanned across multiple frames, effectively incorporated by the bidirectional ConvLSTM into the segmentation process. However, longer sequences do not consistently yield better results, with a peak observed at five frames and subsequent stagnation or decline. While ConvLSTMs theoretically handle

longer sequences, our results suggest that varying the sequence length could be a crucial hyperparameter linked to dataset complexity. For instance, PNS+ [9] utilizes one anchor frame and five randomly selected subsequent frames for the same dataset which is similar to our five frames peak performance.

2.8 Distance between frames

In polyp video segmentation it is common to use five consecutive frames as the input sequence [7–9]. However, at a frame rate of 30 fps the frames might look too similar and contain almost the same information. Larger distances between the input could therefore improve the segmentation results. Table 12 presents the results for *Polyp-NextLSTM* trained and tested with frame distances from one to five, where a frame distance of one means that consecutive frames are used. The sequence length is fixed to five frames. It can be seen that the best results are achieved for a frame distance of one, as used for the training of our proposed model.

Frame	Easy Unseen				Hard Unseen			
Distance	Dice	IOU	HD95	Recall	Dice	IOU	HD95	Recall
1	**0.7686**	**0.6958**	**15.91**	**0.7350**	**0.7838**	**0.7067**	**14.07**	**0.7641**
2	0.7474	0.6755	16.65	0.7059	0.7581	0.6813	15.12	0.7310
3	0.7358	0.6625	18.65	0.6916	0.7338	0.6551	16.64	0.6985
4	0.7333	0.6601	17.96	0.6904	0.7446	0.6659	16.15	0.7126
5	0.7361	0.6638	17.13	0.6924	0.7586	0.6823	14.11	0.7277

Table 12 Test results on the unseen test sets for the ConvNextLSTM model trained and tested with a frame distance of one to five.

3 Results on the PolypGen dataset

We use the PolypGen dataset [15] to validate the generalization capabilities. The PolypGen dataset consists of both, single frames and sequence data. The sequence data is further divided into 23 positive examples (2225 frames) and 23 negative examples (4275 frames). For our tests we only use the positive sequences. In comparison to SUN-SEG, the PolypGen sequences have a visibly lower frame rate that also differs from clip to clip. Furthermore, different from SUN-SEG, the positive sequences also contain various negative frames where no polyp is visible.

Table 13 shows the results of the models trained on SUN-SEG and tested on PolypGen. Generally it can be seen that the performance metrics are considerably worse in comparison to the results on the SUN-SEG test set. However, among all state-of-the-art approaches, our model shows the best performance on the dice score (+0.0061/+1.119%), the IOU (+0.0087/+1.931%) and the Hausdorff distance (-1.23/-3.779%). Only for the recall, SSTAN shows the best results.

		PolypGen					
		Dice	IOU	HD95	Recall	Params	FPS
**Image**	DeepLab [1]	0.5062	0.4376	35.49	0.5314	39.63M	54
	PraNet [2]	0.5137	0.4505	33.32	0.5266	32.55M	45
	SANet [3]	0.4979	0.4288	34.10	0.4990	23.90M	71
	TransFuse [4]	0.4743	0.4122	44.87	0.5162	26.27M	63
	CASCADE [5]	0.5050	0.4414	37.82	0.5321	35.27M	54
**Video**	COSNet [6]	0.4095	0.3512	50.16	0.4370	81.23M	16
	HybridNet [7]	0.4794	0.4053	32.54	0.4882	101.5M	67
	PNSNet [8]	0.4843	0.4162	40.13	0.5183	26.87M	61
	PNS+ [9]	0.5031	0.4381	35.74	0.5263	26.87M	57
	SSTAN [10]	0.4949	0.4310	43.46	**0.5359**	30.15M	101
	**Ours**	**0.5198**	**0.4592**	**31.31**	0.5281	**21.95M**	**108**

Table 13 Results for the models trained on SUN-SEG on the positive sequence data from the PolypGen dataset.

The results show that *PolypNextLSTM* is also able to adapt well to new and completely different data in comparison to other state-of-the-art methods, as it shows the best results for dice score, IOU and HD95. However, the overall performance of all models is rather unsatisfying. A reason for this might be that the SUN-SEG dataset was completely recorded at a single hospital. This might add some intrinsic bias to the data due to the used medical equipment and general procedure which makes it hard for the models to generalize to data from different medical centers.

References

[1] Chen, L., Papandreou, G., Schroff, F., Adam, H.: Rethinking atrous convolution for semantic image segmentation. CoRR **abs/1706.05587** (2017) 1706.05587

[2] Fan, D.-P., Ji, G.-P., Zhou, T., Chen, G., Fu, H., Shen, J., Shao, L.: Pranet: Parallel reverse attention network for polyp segmentation. In: Medical Image Computing and Computer Assisted Intervention – MICCAI 2020, pp. 263–273. Springer, Cham (2020)

[3] Wei, J., Hu, Y., Zhang, R., Li, Z., Zhou, S.K., Cui, S.: Shallow attention network for polyp segmentation. In: International Conference on Medical Image Computing and Computer-Assisted Intervention, pp. 699–708 (2021). Springer

[4] Zhang, Y., Liu, H., Hu, Q.: TransFuse: Fusing Transformers and CNNs for Medical Image Segmentation (2021)

[5] Rahman, M.M., Marculescu, R.: Medical image segmentation via cascaded attention decoding. In: Proceedings of the IEEE/CVF Winter Conference on Applications of Computer Vision (WACV), pp. 6222–6231 (2023)

[6] Lu, X., Wang, W., Ma, C., Shen, J., Shao, L., Porikli, F.: See More, Know More: Unsupervised Video Object Segmentation with Co-Attention Siamese Networks (2020)

[7] Puyal, J.G.-B., Bhatia, K.K., Brandao, P., Ahmad, O.F., Toth, D., Kader, R., Lovat, L., Mountney, P., Stoyanov, D.: Endoscopic polyp segmentation using

a hybrid 2d/3d cnn. In: Medical Image Computing and Computer Assisted Intervention – MICCAI 2020, pp. 295–305. Springer, Cham (2020)

[8] Ji, G.-P., Chou, Y.-C., Fan, D.-P., Chen, G., Fu, H., Jha, D., Shao, L.: Progressively normalized self-attention network for video polyp segmentation. In: International Conference on Medical Image Computing and Computer-Assisted Intervention, pp. 142–152 (2021). Springer

[9] Ji, G.-P., Xiao, G., Chou, Y.-C., Fan, D.-P., Zhao, K., Chen, G., Gool, L.V.: Video polyp segmentation: A deep learning perspective. Machine Intelligence Research **19**(6), 531–549 (2022) https://doi.org/10.1007/s11633-022-1371-y

[10] Zhao, X., Wu, Z., Tan, S., Fan, D.-J., Li, Z., Wan, X., Li, G.: Semi-supervised spatial temporal attention network for video polyp segmentation. In: International Conference on Medical Image Computing and Computer-Assisted Intervention, pp. 456–466 (2022). Springer

[11] Liu, Z., Hu, H., Lin, Y., Yao, Z., Xie, Z., Wei, Y., Ning, J., Cao, Y., Zhang, Z., Dong, L., Wei, F., Guo, B.: Swin Transformer V2: Scaling Up Capacity and Resolution (2022)

[12] Deng, J., Dong, W., Socher, R., Li, L.-J., Li, K., Fei-Fei, L.: Imagenet: A large-scale hierarchical image database. In: 2009 IEEE Conference on Computer Vision and Pattern Recognition, pp. 248–255 (2009). Ieee

[13] Pytorch: Models and pre-trained weights. accessed: 10.10.2023. https://pytorch.org/vision/stable/models.html

[14] Liu, S., Huang, D., Wang, Y.: Receptive field block net for accurate and fast object detection. CoRR **abs/1711.07767** (2017) 1711.07767

[15] Ali, S., Jha, D., Ghatwary, N., Realdon, S., Cannizzaro, R., Salem, O.E., Lamarque, D., Daul, C., Riegler, M.A., Anonsen, K.V., Petlund, A., Halvorsen, P., Rittscher, J., Lange, T., East, J.E.: A multi-centre polyp detection and segmentation dataset for generalisability assessment. Scientific Data **10**(1) (2023) https://doi.org/10.1038/s41597-023-01981-y
